# Endoscopic submucosal dissection combined with endoscopic hand suturing for a laterally spreading tumor spanning the anastomosis after radical resection of a rectal carcinoma

**DOI:** 10.1055/a-2248-0634

**Published:** 2024-03-14

**Authors:** Shibo Song, Yi Liu, Lizhou Dou, Guiqi Wang

**Affiliations:** 1 Department of Endoscopy, National Cancer Center/National Clinical Research Center for Cancer/Cancer Hospital, Chinese Academy of Medical Sciences and Peking Union Medical College, Beijing, China; 2 117892 Endoscopic Center, The First Affiliated Hospital of Xiamen University, Xiamen, China

Precancerous lesions or early cancers located at the anastomosis after radical resection of a rectal carcinoma are relatively rare 1 2 . Recently, several studies have suggested that endoscopic submucosal dissection (ESD) is a favorable treatment for such lesions 3 4 5 ; however, because of severe adhesion in the submucosa of lesions that are located at the anastomosis, partial excision of the circular muscle layer may be required to prevent positive margins. We report a novel measure combining endoscopic hand suturing (EHS) with ESD, to close the postoperative defect following this, which is expected to be an ideal endoscopic method for precancerous lesions or early cancers that span the anastomosis after radical resection of a rectal carcinoma.

A 50-year-old man who underwent radical surgery for rectal cancer 1 year previously was diagnosed on follow-up with a 1.5 × 1.2-cm laterally spreading tumor (LST) spanning the anastomosis (about 5 cm from the anal verge) ( Fig. 1 **a** ). Although the biopsy of lesion showed low grade intraepithelial neoplasia (LGIN), given its malignant potential, the patient underwent ESD. The lesion was removed en bloc with partial muscularis propria to ensure the negative margin ( Fig. 1 **b** ). We sutured the defect with EHS ( Video 1 ). The muscularis propria was also partially sutured to avoid submucosal dead space ( Fig. 1 **c** ). Clips were used to fix the tail and head of the suture ( Fig. 1 **d** ). The resection and suture times were 41 and 30 minutes, respectively. No adverse events occurred. Histologically, complete resection of the LGIN was obtained.

**Fig. 1:**
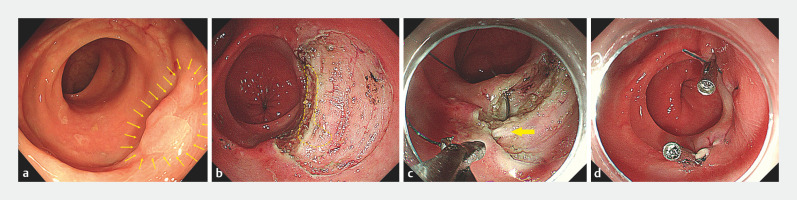
Endoscopic images showing: **a** a 1.5×1.2-cm laterally spreading tumor (yellow arrows) spanning the anastomosis; **b** the defect left after endoscopic submucosal dissection (the yellow circle represents the area where partial muscularis propria was removed); **c** the defect being sutured, with the muscularis propria partially sutured to avoid submucosal dead space (yellow arrow); **d** the closed defect without dehiscence, with clips used to fix the tail and head of the suture.

A rectal defect after endoscopic submucosal dissection with partial excision of the circular muscle layer is completely closed using endoscopic hand suturing.Video 1

The use of ESD combined with EHS to treat an LST spanning the anastomosis has not been previously reported. Despite severe submucosal adhesion at the anastomotic site, it was still possible to remove the lesions en bloc with ESD, thereby preventing the patient needing to undergo further surgery, while EHS effectively prevented any wound-related adverse events. Further clinical experience with this technique is desirable.

Endoscopy_UCTN_Code_TTT_1AQ_2AG

## Notes

Endoscopy E-Videos https://eref.thieme.de/e-videos
*E-Videos* is an open access online section of the journal *Endoscopy* , reporting on interesting cases and new techniques in gastroenterological endoscopy. All papers include a high-quality video and are published with a Creative Commons CC-BY license. Endoscopy E-Videos qualify for HINARI discounts and waivers and eligibility is automatically checked during the submission process. We grant 100% waivers to articles whose corresponding authors are based in Group A countries and 50% waivers to those who are based in Group B countries as classified by Research4Life (see: https://www.research4life.org/access/eligibility/ ). This section has its own submission website at https://mc.manuscriptcentral.com/e-videos .
